# Dairy manure reception pits as reservoirs of extracellular DNA-associated antibiotic resistance genes

**DOI:** 10.1128/spectrum.03688-25

**Published:** 2026-05-22

**Authors:** Najmuj Sakib, Daniel Andersen, Laura Jarboe, Adina Howe

**Affiliations:** 1 Department of Agricultural and Biosystems Engineering, Iowa State University 669136 , Ames, Iowa, USA; 2 Department of Microbiology, Jashore University of Science and Technology 421984, Jashore, Bangladesh; 3 Interdepartmental Microbiology Graduate Program, Ames, Iowa, USA; 4 Department of Chemical and Biological Engineering, Iowa State University 310896 , Ames, Iowa, USA; Connecticut Agricultural Experiment Station, New Haven, Connecticut, USA

**Keywords:** dairy manure, manure pit, exDNA, ARGs, MGEs, antibiotics, resistance

## Abstract

Dairy manure reception pits receive fresh manure and milkhouse wash water, and have short residence times, yet their role as reservoirs of extracellular antibiotic resistance genes (exARGs) remains poorly characterized. Here, we quantified six ARGs (*tetG*, *tetM*, *tetX-*tetracycline, *sul1*-sulfonamide, and *ermB-*macrolide) and three integron-associated mobile genetic elements (MGEs: *intI1*, *intI2*, and *intI3*) in intracellular DNA (iDNA) and extracellular DNA (exDNA) from fresh manure and from a dairy reception pit with an estimated residence time of <6 h. While total DNA yields were lower in the pit relative to fresh manure samples, exDNA-to-iDNA ratios were significantly higher for all genes (*P* < 0.001), indicating rapid enrichment of exDNA after manure entered the pit and mixed with the wash water. Notably, *tetM* exhibited a higher free (unattached) to bound (surface-attached) exDNA ratio in pit samples, which suggested greater accessibility to this gene within the extracellular pool in the pit. Exploratory correlation analysis identified recurring ARG-MGE associations, whereas 16S rRNA gene profiling showed iDNA-associated taxonomic composition diverged between fresh and pit samples, while exDNA-associated taxonomic profiles remained more similar. This result could support that exDNA is relatively stable over time and in varying environments, and that iDNA is more reflective of selective pressures. Together, these results indicate that even short-term reception pits can act as reservoirs of exDNA-associated resistance determinants and justify further work on their persistence, mobility, and management in dairy systems.

**IMPORTANCE:**

To date, extracellular DNA (exDNA) has been shown to contribute to the spread of antibiotic resistance genes (ARGs) in the environment; however, few studies have evaluated its enrichment in short-term dairy pit-stored manure systems. This study demonstrates that dairy manure pits concentrate exDNA during short-term storage and serve as reservoirs for ARGs, along with mobile genetic elements that can facilitate subsequent gene transfer. The results of this study are a strong rationale for further investigation and targeted management strategies of exDNA in manure pits.

## INTRODUCTION

Antibiotic use in livestock is essential for animal health, but it can also increase selective pressure for antibiotic-resistant bacteria and antibiotic resistance genes (ARGs) in the gut and, consequently, in manure (1, 2). The World Health Organization has reported that up to 80% of total antibiotic consumption occurs in the animal sector (3), and this demand is projected to grow globally, as antimicrobial sales are expected to rise 11.5% by 2030 across all continents (4). In the United States’ dairy industry, up to 90% of operations use intramammary antibiotics, especially β-lactams (e.g., penicillins and cephalosporins) and tetracyclines, primarily for dry cow therapy (5). Manure management represents a critical interface between on-farm antibiotic use and environmental dissemination of antimicrobial resistance. Importantly, dairy manure handling is often mediated by multiple interconnected units rather than a single storage unit. In particular, many farms route manure through reception (or blend) pits that collect and mix fresh manure from barn and parlor surfaces with additional liquid streams, such as parlor/milkhouse wash water, before solid–liquid separation and transfer to longer-term storage. Published field studies explicitly distinguish manure reception pits from long-term storage systems when quantifying dairy manure ARG profiles, reflecting that these units can differ in physicochemical conditions, microbial ecology, and temporal variability in ARG abundance (6, 7). In this context, reception pits represent a highly mixed environment with short residence times that may influence ARG risk by rapidly shifting how resistance determinants are partitioned.

Within dairy manure, as well as animal manures broadly, many studies have focused on characterizing AMR risks through the identification and quantification of ARGs in manure and manure pits (5, 8–10). The emphasis of these studies has been on intracellular DNA (iDNA) encoding for ARGs. More recently, extracellular DNA (exDNA) has been identified as playing an increased role in the dissemination of antibiotic resistance (11–13). ExDNA originates from the lysis of dead bacteria or is actively secreted by living cells. Within a microbial community, ARGs can be contained in iDNA, exDNA, or both. Thus, there can be both cell-associated intracellular ARGs (iARGs) and extracellular ARGs (exARGs). ExDNA is generally considered free and readily accessible to competent cells (14, 15), and there are concerns that exARGs may be taken up by competent bacterial cells and genes that encode for antibiotic resistance between bacteria (16).

Beyond their inherent accessibility, the persistence and mobility of exARGs can be influenced by environmental and operational factors within manure systems. For example, dairy cleaning agents, such as disinfectants, can accelerate exDNA release and transformation (17, 18), while mobile genetic elements (MGEs), like integrons, further facilitate gene transfer (19). This suggests that manure pits containing abundant exARGs and MGEs could be hotspots for resistance dissemination. While studies on exARGs in various environments are available, they primarily focus on sludge and compost from livestock (20–26), wastewater (11, 27), manure-amended soil (28, 29), and fertilizers (30, 31). Manure storage pits containing abundant exARGs and MGEs could also serve as hotspots for resistance dissemination. In addition, chemical stressors, such as heavy metals and disinfectant residues, introduced via wash water can promote cell lysis, releasing iDNA into the extracellular pool. Once released, exDNA can bind to organic matter and mineral surfaces in manure, protecting it from enzymatic degradation and allowing exARGs to accumulate relative to iARGs, which are subject to continuous cellular turnover (32, 33).

In this study, we evaluate the hypotheses that short-term exposures to chemical and biological pressures in manure receiving pits result in a greater enrichment of ARGs compared to fresh manure and that this enrichment is greater in exARGs compared to iARGs. To test this hypothesis, samples from fresh manure and the manure-receiving pit at an operational dairy farm were compared. Within each sample, ARGs were classified as iARGs or exARGs based on their location within or external to bacterial cells. In exDNA fractions, we further estimated the quantities of free versus bound fractions of exARGs. We particularly focused on characterizing selected ARGs previously identified in dairy manures, genes associated with resistance to macrolides (*ermB*)*,* sulfonamides (*sul1*)*,* and tetracyclines (*tet33, tetG, tetM, tetX*) (34). We also identified MGEs that may influence ARG transfer, specifically integrons (*intI1, intI2, intI3*). Finally, we compared the taxa found in both iDNA and exDNA fractions from the same sample to better understand the origin of these ARGs. This study is the first to characterize exARGs and iARGs in dairy manure reception pit systems and compare their AMR risks relative to fresh manure.

## MATERIALS AND METHODS

### Site description, sample collection, and processing

Fresh manure was collected in July 2023 from the loafing floor of a dairy (lactating cows) barn located at the Iowa State University Dairy Research Farm (GPS coordinates: 41.9777061–93.6494022). These cows have previously been treated with antibiotics, though no antibiotics were applied to the feed. There were 420 milking cows at the time of sampling; among those, 90.3% were Holstein, and 6%–8% were Jersey. Manure was collected as a slurry sample from a reception pit prior to solid–liquid separation; the pit is located approximately 20 m from the barn and receives manure from pen surfaces and parlor and milkhouse wash water for short-term holding (less than 6 h) prior to downstream processing. For DNA extraction, three biological samples of fresh and pit manure were obtained. Fresh manure was a composite of 3–5 subsamples from different pen areas and contained minimal straw bedding. The solids content for fresh manure and slurry was 12 wt% and 4%, respectively. Samples were then placed in a 500 mL screw-cap plastic container which was filled up to three-fourths full to accommodate any gas creation. A portion of the samples was immediately processed for DNA extraction, and the rest was divided to be kept at 4°C for short-term usage and at –80°C for long-term storage.

### iDNA and exDNA extraction

To separate iDNA and exDNA, a previously described fractionation method (14) was used with modifications. Briefly, samples were subjected to three sequential extractions of increasing stringency: (i) isotonic PBS to recover free (unattached) DNA; (ii) PBS-EDTA to chelate divalent cations and displace weakly surface-bound DNA; and (iii) PBS-trypsin to enzymatically release tightly bound DNA. The remaining cell pellet was then processed separately for iDNA extraction. Full procedural details are provided in Supplementary method M1. After fractionation, DNA was extracted using the DNeasy PowerSoil Pro kit (Qiagen Laboratories, Germantown, MD, USA). To estimate the extraction efficiency for extracellular DNA, a subset of samples were spiked with live whole cells following the protocol of McKinney et al. (29). Briefly, sub-samples of 100 mg (dry weight equivalent) manure (fresh or pit) were spiked with 100 uL whole cells (~10^8^ cfu/mL, OD_600_ = 1.8, 17.5 h overnight culture) of an *Escherichia coli* (*E. coli* MG1655) encoding green fluorescence protein gene (*gfp*) (obtained from Ichiro Matsumura, Addgene plasmid # 26702; https://www.addgene.org/26702/). DNA was extracted with the same methods as described above. Plasmids were extracted using Quick Plasmid Miniprep Kit (Thermo Fisher Scientific, Waltham, MA, USA) and transformed into *E. coli* via electroporation. Quantitative real-time PCR (qPCR) was used to quantify the *gfp* copies in the extracted exDNA. A separate DNA extraction of transformed *E. coli* cells was carried out to quantify the initial *gfp* copies per milliliter of the sample. Extraction efficiency was then calculated as the ratio of *gfp* copies recovered from spiked manure samples to the number of *gfp* copies originally spiked into the samples. Unlike the protocol of McKinney et al. (29), which included DNA spiking, only whole-cell spiking was done for the current study. The DNA extraction yield and quality were verified with spectrophotometry (Nanodrop 2000 and Quant-it dsDNA Assay kit, High sensitivity, Thermo Fisher Scientific) and gel electrophoresis.

### Quantitative real-time qPCR for MGEs and ARGs

All extracted DNA samples were diluted to 1–2 ng/µL so that all measured concentrations fell within the range of known standards. qPCR assays were performed using previously described primers targeting *intI1*, *intI2*, *intI3*, *ermB*, *sul1*, *tet33*, *tetG*, *tetM,* and *tetX* genes (35). The reactions were performed using a 96-well plate on a CFX96 Touch Real-time qPCR detection system (Biorad) using SYBR Green. Templates of DNA standards were synthesized using gBlocks Gene fragments (IDT). Melt curve analysis was performed for all SYBR Green qPCR assays and confirmed the presence of a single, specific amplification product for each target gene. Detailed information on the primers, templates, and thermocycling conditions is listed in Tables S1 to S3.

Standard templates were serially diluted to yield seven sequential 10-fold concentrations and subsequently used for qPCR standard curves (36). Each gene was quantified in technical triplicate (three independent qPCR reactions per extracted DNA sample), as well as a standard curve and a negative control. This technical triplicate quantification was applied across all three biological replicates per sample type. The limit of quantification (LOQ) was defined as the lowest standard concentration (the most diluted) of the linear range of the standard curve (37). In the rare instances where quantifications were observed in the negative controls, their Ct values were at least 3.3 cycles higher than the LOQ Ct values, ensuring they were well outside the quantifiable range (Table S4). All MGE and ARG qPCR results were normalized per gram (g) of dry manure (i.e., absolute abundance). Moisture contents were determined gravimetrically (28) to normalize the gene data per gram (g) of dry manure for both sample types (details in Supplementary method M2).

### 16S rRNA gene amplicon sequencing

16S rRNA gene amplicon sequencing for both types of DNA was performed by Argonne National Laboratory, utilizing an Illumina MiSeq platform with primers 515F and 806R. The sequencing run configuration was 151 bp × 12 bp × 151 bp, including adapter sequences for Illumina flowcell (38–40). Further details are available in Supplementary method M3.

The raw sequences were first processed for data analysis using DADA2 v1.16 to generate high-resolution amplicon sequence variants (ASVs) (41). Taxonomic classification of these ASVs was performed by comparing ASV sequences against the SILVA (v138) database (42). The key DADA2 (v1.16) pipeline parameters are available in Supplementary method M3. The resulting data were further analyzed in R using the Phyloseq package. Alpha diversity was quantified using the Shannon index through the vegan package (43), with differences between DNA types assessed via Wilcoxon rank-sum tests. To quantify the magnitude of differences between groups, the effect size was calculated using Cliff’s delta when necessary. For beta diversity, Bray-Curtis distances were calculated with vegan (43) and evaluated by using permutational multivariate ANOVA with 999 permutations.

### Data analysis and statistics

All analyses were performed using R software (version 4.3.3). Graphs were generated using the ggplot2 package. All data were log-transformed and checked for normality (Shapiro-Wilk test) and equal variance (Levene’s test) to select the appropriate statistical tests for analysis. The gene copy values for free, weakly bound, and tightly bound DNA were combined and averaged to quantify the total extracellular DNA for each sample. For pairwise comparisons, Welch’s *t*-test was employed when data met the normality assumption; otherwise, the non-parametric Wilcoxon rank-sum test was used. To evaluate differences in gene copy numbers, relative abundances, or ratios across different ARGs within fresh manure or pit manure samples, one-way ANOVA was conducted for normally distributed data, while the Kruskal-Wallis test was applied as a non-parametric alternative. In these analyses, gene copy numbers, relative abundances, or ratios served as dependent variables. For all statistical tests, differences were considered significant at *P* < 0.05. Kendall’s Tau correlation test was also performed to assess the relationship between the absolute abundances of ARGs and MGEs. This non-parametric measure was chosen to account for potential non-linear relationships and to handle tied ranks in the data. Benjamini-Hochberg FDR correction was applied to Kendall’s Tau correlation *P* values to account for multiple comparisons. To maximize statistical power given the limited within-group sample size (*n* = 3 per group), data from fresh and pit manure samples were combined for Kendall’s Tau correlation analyses.

## RESULTS

### Manure reception pit depletes iDNA relative to exDNA

Fresh manure and manure pit samples were taken from an active dairy farm, and iDNA and exDNA were extracted. The extracted iDNA yields ranged from 23.9 to 68.3 µg/g of sample dry weight, whereas exDNA yields—derived from free, weakly bound, and tightly bound fractions—were lower (0.8–10.4 µg/g). DNA yield and purity metrics for all iDNA and exDNA extractions are provided in Table S5. Both iDNA and exDNA were less abundant in pit samples compared to fresh manure (Table 1). To validate the exDNA extraction method, known quantities of whole cell *E. coli* carrying the *gfp* gene were spiked into samples. A low recovery of the *gfp* gene in the exDNA pool (0.003%–0.02%, *P* < 0.05, one-sample *t*-test) indicated that the extraction method effectively minimizes cell lysis and extraction of intracellular DNA.

**TABLE 1:** Extracted yields (ug/g, mean ± SD) of exDNA and iDNA

DNA types	Subtypes	Extracted yields (ug/g)	
		Fresh	Pit
exDNA		11.91 ± 2.10	4.47 ± 0.58
	Free	2.66 ± 0.11	2.59 ± 0.48
	Weakly bound	7.30 ± 2.06	0.86 ± 0.15
	Tightly bound	1.95 ± 0.36	1.01 ± 0.28
iDNA		62.94 ± 6.48	28.26 ± 4.15

**Fig 1:**
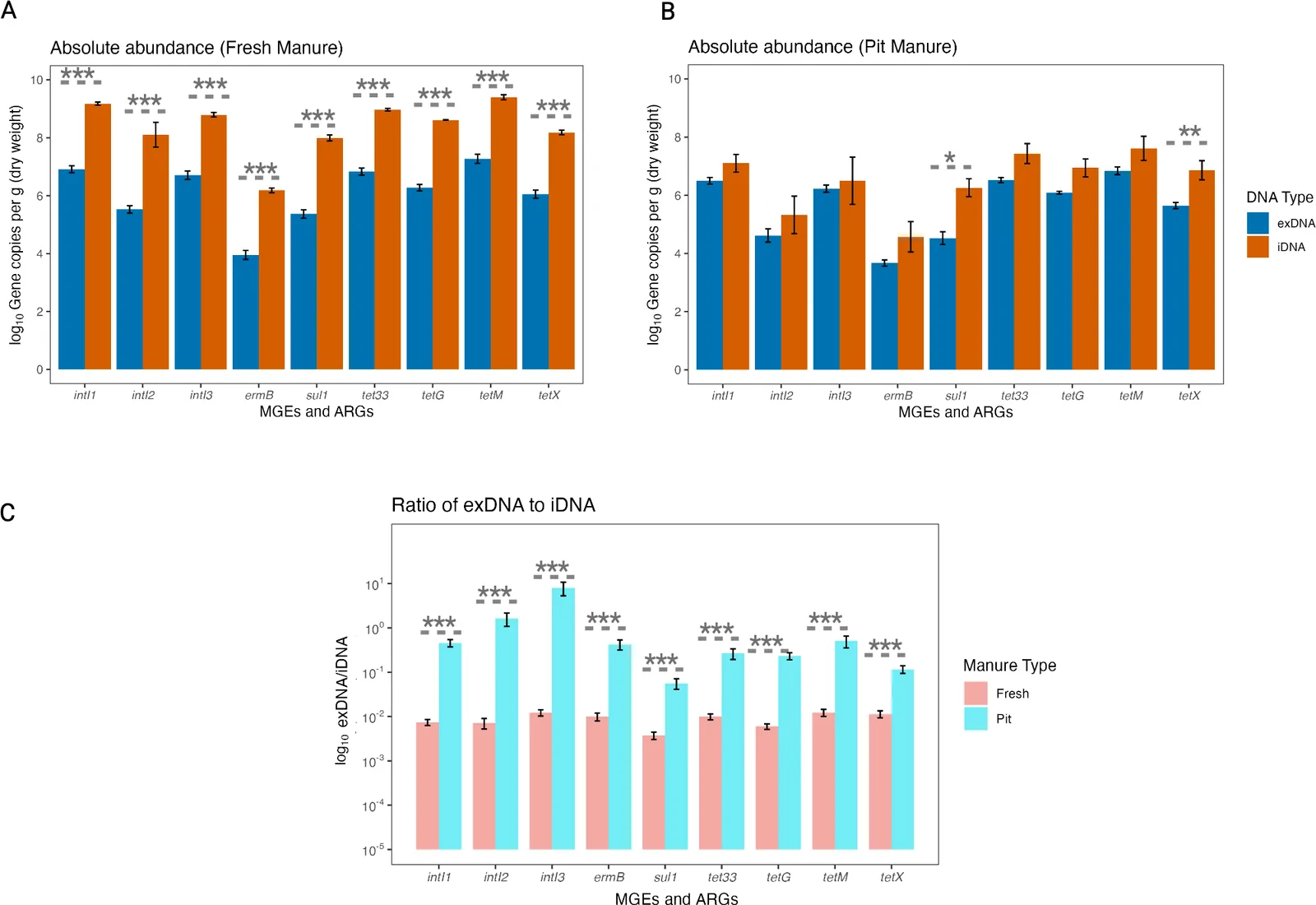
Manure storage pit depletes iDNA genes relative to exDNA. (**A**) Absolute abundance of ARGs and MGEs’ copies per gram dry weight for fresh manure. (**B**) Absolute abundance of ARGs and MGEs’ copies per gram dry weight for pit manure. (**C**) Bar plot showing the ratio of exDNA to iDNA on a log scale. Depending on the normality, pairwise significance between the DNA types (exDNA vs iDNA) or the manure types (fresh vs pit) was determined by an independent sample *t*-test or a Wilcoxon sum rank test. Asterisks indicate statistically significant correlations of *P < 0.05, **P < 0.01, and ***P < 0.001, respectively. Error bars represent standard errors of the means (mean ± SE).

Next, we quantified the abundances of six ARGs (*ermB, sul1, tet33, tetG, tetM, tetX*) and three MGEs (*intI1, intI2, intI3*) in all exDNA and iDNA extractions (Fig. 1). Significant differences between iDNA and exDNA concentrations in all genes were observed in fresh manure (Fig. 1A), with iDNA levels consistently higher than exDNA (*P* < 0.05). Similar trends were observed in pit manure, though only *sul1* and *tetX* genes were significantly more abundant as iDNA relative to exDNA (Fig. 1B). To facilitate a direct comparison between solid fresh manure and slurry pit samples, we calculated the exDNA-to-iDNA ratios for each gene (Fig. 1C). These ratios were significantly higher in pit than in fresh manure across all genes (Wilcoxon rank sum, *P* < 0.001), supporting that exDNA is found at higher proportions than iDNA in pit relative to fresh manure samples.

We quantified both free and bound exDNA fractions within the extracted exDNA pool (Table 1) and calculated the free-to-bound ratio for fresh and pit samples. For different ARGs, these ratios varied across samples, with no consistent patterns. Significant variations (Wilcoxon rank sum, *P* < 0.01) were found for several genes. Specifically, the ratio was higher in pit manure for *tetM* and, to a lesser extent, *intI2,* while it was higher in fresh manure for *intI3* and *tet33* (Fig. S1). The *tetM* was the most prevalent (*P* < 0.001) in free form in the pit relative to fresh manure.

### Correlation analysis shows strong associations between MGEs and ARGs in DNA categories

To explore the relationship between MGEs and ARGs, we classified detected genes into intracellular (iARGs, iMGEs) and extracellular (exARGs, exMGEs) categories based on their presence in iDNA or exDNA fractions.

**Fig 2:**
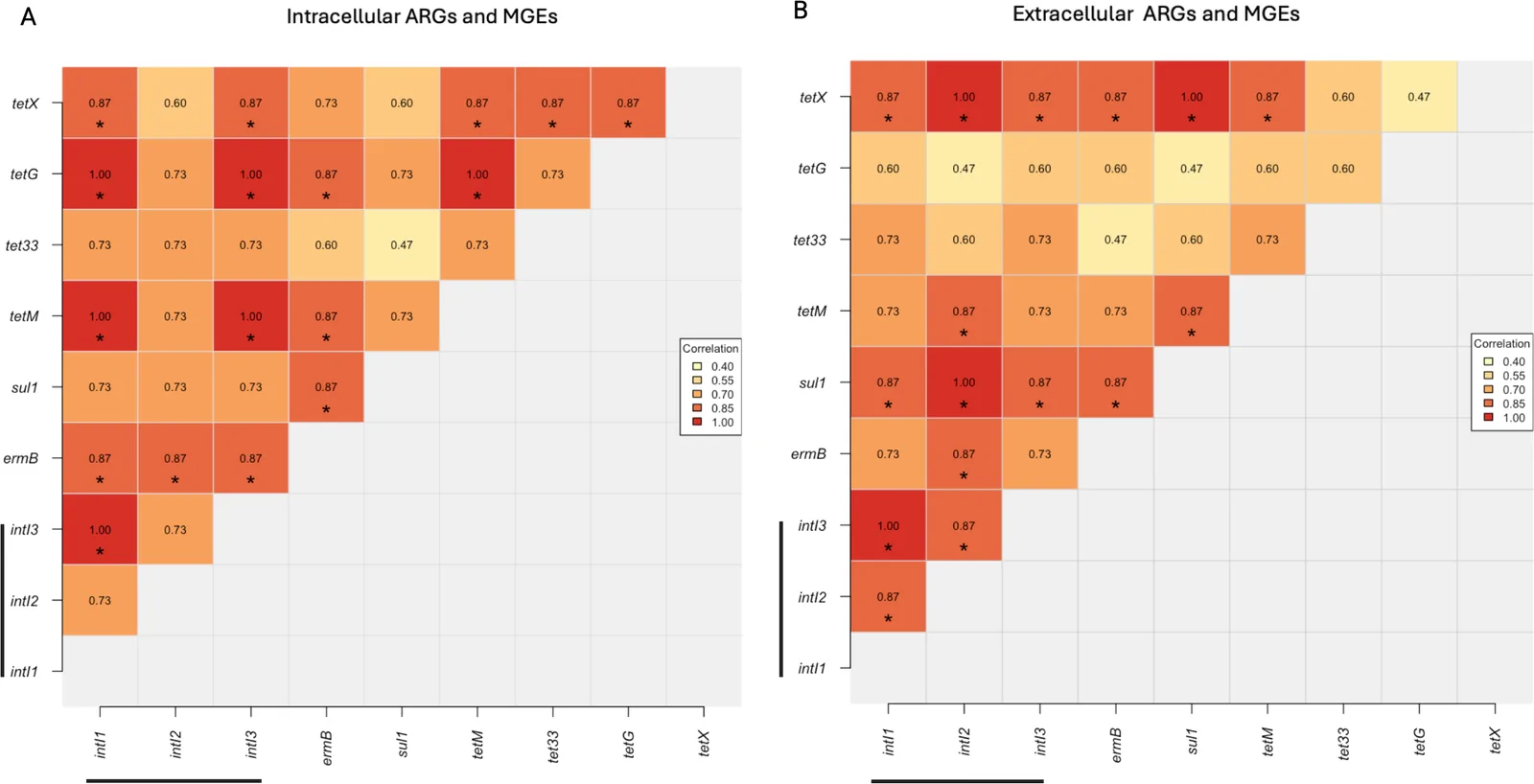
Correlation analysis shows strong associations between MGEs and ARGs in DNA categories. (**A**) Heat map showing the correlation between iARGs and iMGEs (underlined). (**B**) Heat map showing the correlation between eARGs and eMGEs (underlined). Values in each square were determined by Kendall’s Tau correlation coefficient. Asterisks indicate statistically significant correlations after FDR corrections (* for *P* < 0.05).

To increase statistical power and better understand broader patterns of association, data from both fresh and pit samples were combined in subsequent analyses. With the combined data, correlation analysis was used to explore potential relationships between MGEs and ARGs, which may reflect gene mobility or shared selection pressures. Strong associations were observed between iMGEs and iARGs in the iDNA fraction (Fig. 2A), particularly among tetracycline resistance genes (*tetX, tetG, tetM*). Notably, *intI1* and *intI3* were strongly correlated with *tetX, tetG, tetM,* and *ermB*, while *intI2* showed a distinct association with *ermB*. In addition, *tet* genes exhibited strong correlations with one another. In the exDNA fraction (Fig. 2B), *intI1* and *intI3* were linked to *tetX* and *sul1*, whereas *intI2* exhibited broader associations with *tetX, sul1, tetM,* and *ermB*. Interestingly, *sul1* was exclusively correlated within the exMGEs. Across both intracellular and extracellular environments, only four correlations were consistently significant, including *intI1–intI3, intI1–tetX, intI2–ermB, and ermB–sul1*.

### Contrasting compositional profiles between exDNA and iDNA fractions

**Fig 3:**
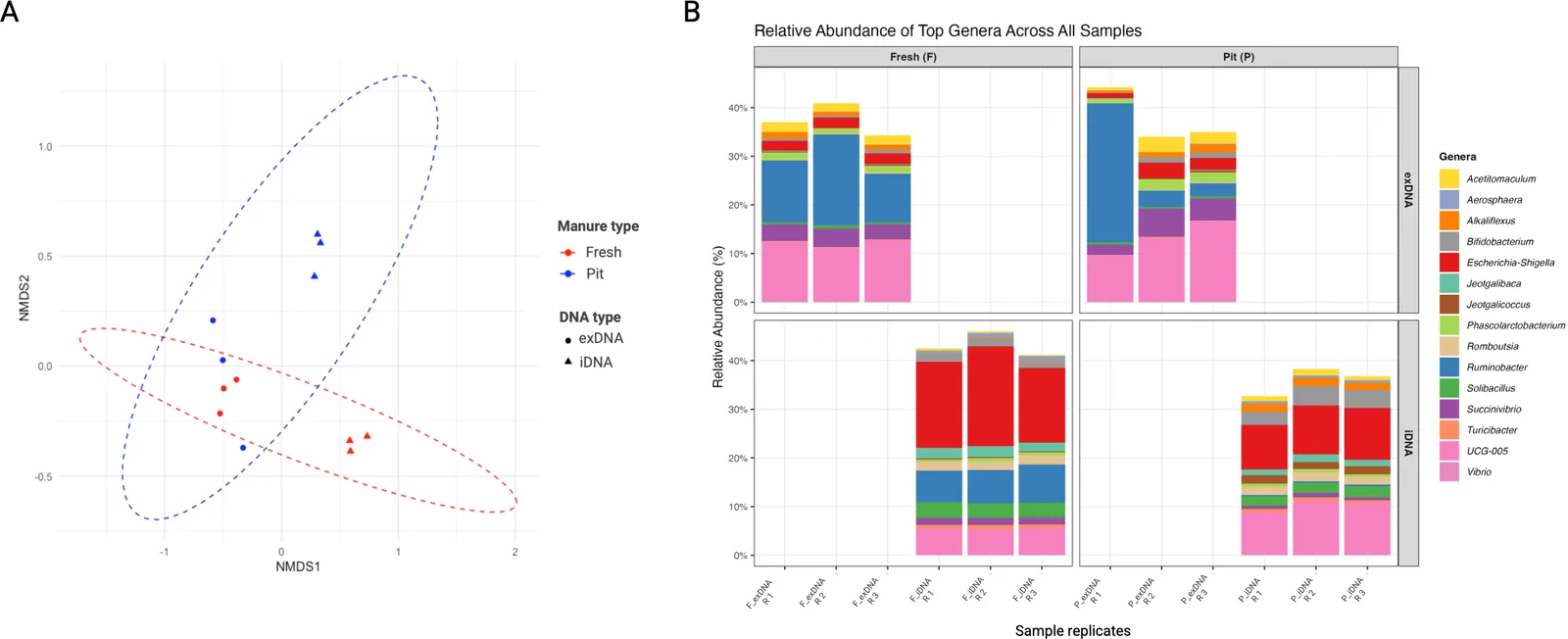
Compositional analysis shows intermixed exDNA profiles but distinct iDNA community structures. (**A**) NMDS plot of Bray-Curtis dissimilarity index for taxa abundances between all sample types. Dashed ellipses represent 95% confidence ellipses for each manure type group (fresh and pit). Significant clustering was analyzed by PERMANOVA (*P* = 0.001, R² = 63.96%). (**B**) Relative abundance of bacterial genera in exDNA and iDNA. The relative abundance equals the number of reads identified for a specific taxon by the total number of reads.

To further investigate the compositional profiles of exDNA and iDNA, we performed 16S rRNA gene amplicon sequencing to characterize the taxa present across both manure types and DNA categories. Shannon diversity was calculated at the phylum level to assess overall microbial community complexity across sample groups. No significant difference was found between fresh and pit samples (Fig. S2A). Both fresh and pit manure were dominated by Proteobacteria, Firmicutes, and Bacteroidetes. Other phyla, such as Actinobacteria, Spirochaetes, and Verrucomicrobia, were also detected but in lower abundance (Fig. S2B and S3A). We also performed non-metric multidimensional scaling (NMDS) analysis (stress value of 0.06, Fig. 3) to assess compositional differences between manure types and DNA fractions. The analysis revealed distinct clustering of iDNA communities from fresh and pit manure, supported by a significant PERMANOVA result (*P* = 0.001, R² = 63.96%) and indicates strong compositional differences. These differences were further supported at the genus level (Fig. 3B; see also Fig. S3B); iDNA from fresh manure included genera, such as *Ruminobacter*, whereas pit manure was enriched in genera like *Bifidobacterium*. In contrast, taxa present in exDNA fractions from both manure types clustered together, suggesting greater similarity in extracellular DNA compositional profiles across manure types. The homogeneity of the dispersion was not significantly different between fresh and pit manures (*P* = 0.75), suggesting that results are likely due to true differences in microbial community composition rather than differences in within-group variability.

## DISCUSSION

This study addresses a critical gap in understanding how exDNA, particularly ARGs within exDNA, change during the storage of dairy manure and wash water in a short-residence reception pit. Our results indicate exDNA is present in both fresh and pit-stored manures. We find that iDNA consistently exceeded exDNA in abundance across all manure samples, consistent with patterns observed in other nutrient-rich, high-microbial-density environments (11, 20). However, gene-specific exDNA-to-iDNA ratios revealed relatively higher exDNA levels in pit-stored samples. This enrichment of ARGs within exDNA suggests that even short-term manure storage may facilitate the extracellular accumulation of genetic material encoding for antibiotic resistance determinants.

Several factors may contribute to the enrichment of exDNA in manure pits on short timescales. Reception pits are characterized by rapid mixing and repeated loading, which may create conditions that promote microbial cell lysis, releasing intracellular DNA into the extracellular environment. The use of chlorinated disinfectants in dairy wash water can further accelerate this process by altering ionic strength, inducing oxidative stress, and compromising cell membranes, leading to increased DNA release (44–46). Additionally, the anaerobic and nutrient-rich conditions in pits may slow down DNA degradation by limiting nuclease activity, allowing exDNA to persist for extended periods (14, 33). At the same time, reception pits can lower apparent iDNA concentrations through the dilution of biomass when additional liquid streams reduce solids content, even if absolute exDNA production is modest. Once released, exDNA can rapidly partition into different states; adsorption and cation bridging to minerals and organic matter can begin stabilizing DNA soon after release, potentially protecting fragments from enzymatic degradation and altering the balance between extracellular and intracellular pools (32, 33, 47, 48). Thus, a reception pit can act as an early sorting environment in which short-term physicochemical conditions shape whether resistance determinants are detected predominantly as iARGs (cell-associated) or exARGs (extracellular and potentially more mobile), without requiring long-term residence.

The physicochemical properties of dairy manure likely govern the retention, protection, and mobilization of ARGs via exDNA, as reflected by the varying free-to-bound ratios observed in the samples. Pit samples showed greater ratios of free-to-bound exDNA specifically for *tetM*. This pattern suggests that *tetM* may be more prevalent in a free and potentially more accessible form compared to its presence in fresh manure samples. This availability in dairy pits could potentially increase its association with horizontal gene transfer. Conversely, genes, such as *intI2*, *intI3*, and *tet33,* were more bound in pit samples than in fresh manure. Together, these observations suggest that ARGs may vary in their potential to be in extra- or intracellular DNA pools. The degree to which an ARG is bound in DNA pools can also affect the persistence of these determinants in the environment, as manure is applied to agricultural soils. Previous studies have shown that exDNA can persist in soils for extended periods and that transformation can occur rapidly under favorable conditions, even with relatively low exDNA concentrations (26, 32, 49, 50), and thus our results present a strong rationale for further study on the enrichment of exDNA in manure pits and its persistence both in the pit and downstream in land application.

Our observation that integrons were enriched in exDNA in pit manures is significant because these genes are often embedded within mobile elements (51, 52). While they typically reside on chromosomes, environmental pressures from pollutants like antibiotics and heavy metals can force their mobilization, especially on plasmids (19). In our study, *intI1* was found to have the highest absolute abundance among integron genes. This observation aligns with previous findings reporting *intI1* as abundant in both exDNA and iDNA copies in various anthropogenic and agricultural waste sources (53, 54). The persistence of extracellular *intI1* has also been abundant in samples from wastewater treatment plants (55). Moreover, our study found *sul1* associated with integrons in exDNA. This finding is significant given that *sul1* is often part of the 3′-conserved segment of class 1 integrons (9, 56). Its presence in exDNA suggests a potential for horizontal gene transfer via plasmids and is consistent with previous studies showing that *sul1*-containing plasmids (e.g., IncF plasmid) can be conjugally transferred (56). Recent research also identified an increase in *sul1* in the exDNA pool across wastewater treatment plant systems (57). In iDNA pools, we observed a correlation between *intI2* and *ermB*. This association implies that *ermB*, which confers resistance to macrolide antibiotics, may be co-selected with integrons. The association of *ermB* with integrons (i.e., *intI1*) has also been previously reported in freshwater iDNA samples (58). The presence of this co-located arrangement in iDNA and not exDNA suggests that the genetic linkage is actively maintained within living bacteria (59). This is mechanistically consistent with the nature of each DNA fraction: in iDNA, ARGs and MGEs reside on the same intact chromosome or plasmid within a living cell, preserving their physical co-location and co-inheritance. In contrast, exDNA consists of fragmented DNA released upon cell lysis, which can disrupt the detectable co-occurrence of elements that were originally genomically linked. Furthermore, exDNA fractions may contain DNA from multiple organisms and lysis events, further obscuring specific ARG-MGE linkages. It should be noted, however, that the correlation analyses underpinning these observations were conducted on pooled data from fresh and pit manure samples, which was necessary to achieve sufficient statistical power given the small within-group sample size. As a result, some of the observed ARG-MGE associations may reflect systematic differences between manure types rather than direct biological co-variation within a single environment.

Understanding the compositional profiles of exDNA and iDNA fractions provides an additional context for interpreting ARG dynamics and their potential for horizontal transfer. Although the 16S rRNA gene is typically associated with intact bacterial cells, its detection in exDNA is common and may result from recent cell lysis, active secretion as part of biofilm formation, or other physiological processes such as outer membrane vesicle release (53, 54, 57). Our results indicate distinct beta diversity patterns across DNA pools from fresh and pit manure sources. While both fractions share similar taxa, their overall community structures differ substantially. This result likely reflects differences in selective pressures, which are consistently acting on iDNA from living cells, but not on exDNA, which comes from lysed cells and is less affected by the surrounding environmental forces (60). The relative similarity of exDNA profiles across manure types suggests that extracellular pools may get contributions from multiple upstream sources and may respond more slowly (or differently) than iDNA to immediate selective pressures. This aligns with prior work showing that exDNA can “mask” or dilute iDNA-derived signals in sequencing-based studies of anaerobic systems (61). Hence, it reinforces the value of separating iDNA from exDNA when interpreting microbiome and resistome patterns. On the other hand, the differences in iDNA between fresh and pit manure reflect their distinct environments. In our study, we detected enriched *Ruminobacter* in fresh and *Bifidobacterium* in the pit iDNA. These results support that fresh manure originates from the gut of the animal and is fiber-rich, as we see it supporting genera like *Ruminobacter* that thrive on rumen fermentation. In contrast, the pit manure goes through fermentation and storage, creating conditions that encourage the growth of more adaptive genera like *Bifidobacterium* (62, 63). Conversely, exDNA pools from both manure types tend to be more similar, likely because exDNA binds to organic matter, which protects it, preserving a type of historical snapshot of the microbial community that may be less affected by ongoing selective pressures (61).

Although our findings provide important initial insights into exDNA pools in dairy manures, several limitations should be acknowledged. We could not completely eliminate iDNA contamination from our exDNA; however, minimal false positives (gfp recovery of 0.003%–0.02%) were lower than the 1.3% reported previously (29). The presence of the *gfp* gene in exDNA fractions from whole cells of *E. coli* containing the plasmid is likely due to excreted DNA from live or partially lysed cells. Furthermore, the laboratory *E. coli* MG1655 strain used as the spike-in control may not fully represent native manure microbes, which differ considerably in cell wall composition, biofilm-forming capacity, and degree of physical association with manure particles. These differences could affect its susceptibility to lysis during the fractionation procedure, meaning our extraction efficiency estimates may underestimate the degree of indigenous cell lysis that occurs in complex manure matrices. Other studies that used membrane filters to remove microbial cell contamination from exDNA primarily focused on water samples (64–66). In contrast, our study used manure samples, which could trap exDNA on the filters during the filtration step; therefore, this method was avoided. We also acknowledge that the limited biological sample size in this study was a constraint. From a statistical standpoint, this small sample size reduced power to detect true differences between groups, increased the risk of false negatives, and limited confidence in the correlation network structure, which may not have been stable across alternative sample draws. Future research should include a larger number of samples collected across multiple farms, locations, and time points to improve representativeness.

### Conclusion

This study provides new insights into the dynamics of exDNA and its ARGs during dairy manure storage. We demonstrate that manure pits can act as reservoirs for exDNA and suggest that storage conditions, including physicochemical properties and microbial processes, may influence the persistence and potential mobility of ARGs. The enrichment of exDNA in pits raises important questions about its role in horizontal gene transfer in both the pit and manure land application, particularly given the presence of mobile genetic elements, such as integrons and plasmids, and their association with exARGs. Addressing these knowledge gaps through future studies is essential to deepen our understanding of exDNA dynamics and ARG mobility under varying storage conditions and management practices.

## Data Availability

All data are available in GitHub through Zenodo (https://doi.org/10.5281/zenodo.19443820). The sequencing data generated in this study have been deposited in the NCBI Sequence Read Archive (SRA) under BioProject accession number PRJNA1357369.

## SUPPLEMENTAL MATERIAL

Supplemental material spectrum.03688-25-s0001.docx Methods S1–S4, Figures S1–S3, and Tables S1–S5.

ASM does not own the copyrights to Supplemental Material that may be linked to, or accessed through, an article. The authors have granted ASM a non-exclusive, world-wide license to publish the Supplemental Material files. Please contact the corresponding author directly for reuse.
