# Supplementary material for: Dairy manure reception pits as reservoirs of extracellular DNA-associated antibiotic resistance genes
Source: Microbiol Spectr. 2026 May 22;14(7):e03688-25. doi: 10.1128/spectrum.03688-25 (PMC13340301; doi:10.1128/spectrum.03688-25)
Supplement: Supplemental material — Methods S1–S4, Figures S1–S3, and Tables S1–S5. [file spectrum.03688-25-s0001.docx]

***Supplementary Information***

**Dairy manure reception pits as reservoirs of extracellular DNA-associated antibiotic resistance genes**

**Najmuj Sakib**1,3,4**, Daniel Andersen**1**, Laura Jarboe**2,4**, Adina Howe**1,4*

1 Department of Agricultural and Biosystems Engineering, Iowa State University, Ames, IA 50011, United States

2 Department of Chemical and Biological Engineering, Iowa State University, Ames, IA 50011, United States

3 Department of Microbiology, Jashore University of Science and Technology, Jashore 7408, Bangladesh

4 Interdepartmental Microbiology Graduate Program, Ames, IA 50011, United States

* Corresponding Author:

**Adina Howe**

Department of Agricultural and Biosystems Engineering,

Iowa State University, Ames, IA 50011, United States.

Email: adina@iastate.edu

Number of Pages: 12

Number of Tables: 5

Number of Figures: 3

**Supplemental Method**

**M1. Sequential DNA fractionation method**

Extracellular DNA was extracted from dairy manure samples using a modified protocol from Nagler et al., 2018 designed to minimize cell lysis and separate exDNA into three fractions: free DNA (fDNA), weakly bound DNA (wbDNA), and tightly bound DNA (tbDNA) (1). Prior to the extraction procedure, several solutions were prepared. A 5 mM PBS-EDTA stock solution was created by making a 10 mM EDTA-PBS stock (0.8 L of 1X PBS mixed with 20 mL of 0.5 M EDTA, pH adjusted to 6.0, and volume brought to 1 L), and diluting it 1:1 with PBS. PBS-Trypsin (0.125%) and PBS-Trypsin-Inhibitor (0.125%) solutions were prepared by dissolving 0.125 g of the respective compound in 100 mL PBS, followed by sterile filtration (0.22 μm).

For each sample, 100 mg (wet weight) of solid manure or 100 μL of liquid/slurry was processed. To assess potential contamination from intracellular DNA due to cell lysis, an extraction control was included by adding 100 μL (1:1 ratio) of GFP-containing live *E. coli* (OD=1.8, 17.5 hours of overnight culture) to the sample. To extract fDNA, samples were mixed with 700 μL (for solid) or 600 μL (for slurry) of 10x PBS (0.1 M) and agitated at 30 rpm for 1 hour at 4°C. After centrifugation (5000g, 10 min), the supernatant containing fDNA was collected.

The remaining pellet was subjected to mild washing to extract wbDNA using the prepared 5 mM PBS-EDTA solution. Nine volumes of this solution were added to the pellet, gently mixed, and incubated for 5 minutes at room temperature. Following centrifugation (5000g, 10 min, 4°C), the supernatant containing wbDNA was collected. For tbDNA extraction, the pellet was treated with the prepared PBS-trypsin solution for 5 minutes at room temperature, followed by the addition of an equal volume of PBS-trypsin inhibitor solution. After incubation and centrifugation, the supernatant containing tbDNA was collected.

All exDNA fractions were further purified using the DNeasy PowerSoil Pro Kit (QIAGEN), following the manufacturer's instructions (DNeasy® PowerSoil® Pro Kit Handbook: Updated on 06/2023) with modifications. No bead-containing tubes were used to extract exDNA. Hence, the bead-beating step (homogenizing samples with a vortex adapter) was omitted to minimize cell lysis (if whole cells still existed along with exDNA) or exDNA fractionations, and buffers CD1, CD2, and CD3 volumes were adjusted based on previous supernatant volumes. Instead of the provided 2 mL tubes, larger 5 mL sterile tubes were used to hold the volumes. All the excess volumes were also passed through the spin column filter (according to step 13 from the manufacturer’s instruction). The remaining cell pellet was processed separately to extract intracellular DNA (iDNA) following the manufacturer's protocol of the same kit. Final elution volumes were 20-25 μL for exDNA fractions and 50 μL for iDNA to optimize DNA yield for downstream applications.

Although we did not perform any additional wash of the cell pellet (e.g., with PBS) prior to iDNA extraction, this would further minimize potential cross-contamination and is recommended in future iterations of this protocol.

**M2. Gravimetric moisture content determination and gene data normalization**

To determine moisture content gravimetrically and normalize gene data per gram of dry manure, a standardized drying method was employed for both fresh manure and pit manure slurry samples. A clean, pre-weighed container was used for each sample type to hold a known amount of manure. Fresh manure samples were weighed directly, while a known volume of pit manure slurry was measured and its initial weight recorded. All samples were then dried in an oven at 105°C until they reached a constant weight, typically requiring 24-48 hours for fresh manure and potentially longer for slurry samples. After drying, the samples were reweighed to determine the dry matter content. Moisture content was calculated as the percentage of weight lost during drying, and dry matter content was derived as the complement of this value. For pit manure slurry, we additionally calculated the density by dividing the initial wet weight by the known volume. To normalize gene copy numbers, we divided the total gene copies by the dry matter fraction for fresh manure samples. For pit manure slurry, we first converted the sample volume to its equivalent dry weight using the calculated density (i.e., Mass/volume) and dry matter content, then divided the gene copy number by this dry weight. This approach allowed us to express all gene abundance data in terms of copies per gram of dry manure, ensuring comparability between the two sample types despite their different physical states.

From this gene data, the exDNA to iDNA ratio was calculated by combining all exDNA fractions (fDNA, wbDNA and tbDNA) and dividing by the corresponding iDNA fraction across the manure types for a direct comparison. Additionally, the distribution of specific genes in each exDNA fraction was assessed by calculating their percentages separately for fresh and pit manure samples.

**M3. 16S rRNA amplicon sequencing**

Each 25 µL PCR reaction contained 9.5 µL of PCR-grade water (Certified DNA-Free), 12.5 µL of QuantaBio’s AccuStart II PCR ToughMix (2x concentration, 1x final), 1 µL Golay barcode tagged Forward Primer (5 µM concentration, 200 pM final), 1 µL Reverse Primer (5 µM concentration, 200 pM final), and 1 µL of template DNA. The conditions for PCR were as follows: 94°C for 3 minutes to denature the DNA, with 35 cycles at 94°C for 45 s, 50°C for 60 s, and 72°C for 90 s; with a final extension of 10 min at 72°C to ensure complete amplification. Amplicons were then quantified using PicoGreen (Invitrogen) and a plate reader (Infinite 200 PRO, Tecan). Once quantified, volumes of each of the products were pooled into a single tube so that each amplicon was represented in equimolar amounts. This pool was then purified using AMPure XP Beads (Beckman Coulter), and then quantified using a fluorometer (Qubit, Invitrogen). After quantification, the molarity of the pool was determined and diluted down to 2 nM, denatured, and then diluted to a final concentration of 6.75 pM with a 10% PhiX spike for sequencing on the Illumina MiSeq.

The key parameters used in the DADA2 pipeline were as follows. Quality filtering and trimming was performed using filterAndTrim() with truncLen=c(140,140) to truncate both forward and reverse reads at 140 bp, maxN=0 to remove reads with ambiguous bases, maxEE=c(2,2) to discard reads exceeding 2 expected errors in either direction, truncQ=2 to truncate reads at the first base with quality score below 2, and rm.phix=TRUE to remove PhiX spike-in reads. Error rates were learned using learnErrors() with default parameters separately for forward and reverse reads. Sample inference was performed using the dada() function with default settings (independent sample processing, pool=FALSE). Forward and reverse denoised reads were merged using mergePairs() with default parameters (minimum overlap of 12 bp, zero mismatches allowed in the overlap region). Chimeric sequences were identified and removed using removeBimeraDenovo() with method="consensus". Taxonomic classification of ASVs was performed using assignTaxonomy() against the SILVA v138 reference database.

**Supplemental Table S1.** qPCR primers

| Primer | Gene targeted | Primer sequence (5’-3’) | Target size (bp) | Annealing temperature (°C) | Reference |
| --- | --- | --- | --- | --- | --- |
| *intI1F165_clinical_F* | *intI1* | CGAACGAGTGGCGGAGGGTG | 312 | 60 | (2) |
| *intI1R476_clinical_R* |  | TACCCGAGAGCTTGGCACCCA |  |  |  |
| *intI2-F* | *intI2* | TGCTTTTCCCACCCTTACC | 195 | 58 | (2) |
| *int2-R* |  | GACGGCTACCCTCTGTTATCTC |  |  |  |
| *int3-F* | *intI3* | CAGGTGCTGGGCATGGA | 257 | 58 | (2) |
| *int3-R* |  | CCTGGGCAGCATCACCA |  |  |  |
| *erm(B)_F* | *ermB* | GAACACTAGGGTTGTTCTTGCA | 120 | 58 | (2) |
| *erm(B)_R* |  | CTGGAACATCTGTGGTATGGC |  |  |  |
| *sul1-F* | *sul1* | GCCGATGAGATCAGACGTATTG | 102 | 58 | (2) |
| *sul1-R* |  | CGCATAGCGCTGGGTTTC |  |  |  |
| *tet33*-F | *tet33* | TGCTTGTTTCCCTGGCCG | 147 | 58 | This study |
| *tet33*-R |  | GCGTGATGTCGGCGATCA |  |  |  |
| *tetG*-F | *tetG* | CCGCGAGCGACAAACCA | 246 | 58 | (2) |
| *tetG*-R |  | TCGCGTTCCTGCTTGCC |  |  |  |
| *tetM*-F | *tetM* | GGAGCGATTACAGAATTAGGAAGC | 158 | 58 | (2) |
| *tetM*-R |  | TCCATATGTCCTGGCGTGTC |  |  |  |
| *tetX*-F | *tetX* | AAATTTGTTACCGACACGGAAGTT | 101 | 58 | (2) |
| *tetX*-R |  | CATAGCTGAAAAAATCCAGGACAGTT |  |  |  |
| *gfp_F* | *gfp* | ACGACGGCAACTACAAGACC | 94 | 58 | This study |
| *gfp_R* |  | TCCTCCTTGAAGTCGATGCC |  |  |  |

**Supplemental Table S2.** Standard sequences for each primer set with coefficient of determination (r^2^) and amplification efficiency.

| **Gene** | **Standard Length (bp)** | **r^2^** | **Efficiency (%)** | **NCBI Accession Number** | **Standard Sequence** |
| --- | --- | --- | --- | --- | --- |
| 16S rRNA | 500 | 0.99 | 91.00 | LC523634.1 | TGCGAAAGCGTGGGGAGCAAACAGGATTAGATACCCTGGTAGTCCACGCCGTAAACGATGTCGACTTGGAGGTTGTGCCCTTGAGGCGTGGCTTCCGGAGCTAACGCGTTAAGTCGACCGCCTGGGGAGTACGGCCGCAAGGTTAAAACTCAAATGAATTGACGGGGGCCCGCACAAGCGGTGGAGCATGTGGTTTAATTCGATGCAACGCGAAGAACCTTACCTGGTCTTGACATCCACGGAAGTTTTCAGAGATGAGAATGTGCCTTCGGGAACCGTGAGACAGGTGCTGCATGGCTGTCGTCAGCTCGTGTTGTGAAATGTTGGGTTAAGTCCCGCAACGAGCGCAACCCTTATCCTTTGTTGCCAGCGGTCCGGCCGGGAACTCAAAGGAGACTGCCAGTGATAAACTGGAGGAAGGTGGGGATGACGTCAAGTCATCATGGCCCTTACGACCAGGGCTACACACGTGCTACAATGGCGCATACAAAGAGAAGCGA |
| *intI1* | 371 | 0.99 | 89.00 | CP166713.1 | ACTGTCAAGCTTAGCCAATACTGGTTGAGGCGAACGAGTGGCGGAGGGTGTGCGGTGTGGCGGGCTTCGTGATGCCTGCTTGTTCTACGGCACGTTTGAAGGCGCGCTGAAAGGTCTGGTCATACATGTGATGGCGACGCACGACACCGCTCCGTGGATCGGTCGAATGCGTGTGCTGCGCAAAAACCCAGAACCACGGCCAGGAATGCCCGGCGCGCGGATACTTCCGCTCAAGGGCGTCGGGAAGCGCAACGCCGCTGCGGCCCTCGGCCTGGTCCTTCAGCCACCATGCCCGTGCACGCGACAGCTGCTCGCGCAGGCTGGGTGCCAAGCTCTCGGGTATCCTTCTTCAACAATCAGAAGCTTACTGTC |
| *intI2* | 250 | 0.95 | 98.00 | CP166713.1 | ACTGTCAAGCTTCCTGCAAGCAAGCCTAGACGGCTACCCTCTGTTATCTCTGCAAATGAAGTGCAACGCATTTTGCAGGTTATGGATACTCGCAACCAAGTTATTTTTACGCTGCTGTATGGTGCAGGTTTGCGCATTAATGAATGCTTGCGTTTGCGGGTTAAAGATTTTGATTTTGATAATGGCTGCATCACTGTGCATGACGGTAAGGGTGGGAAAAGCAGAAACAGCCTACTGCCAAGCTTACTGTC |
| *intI3* | 500 | 0.99 | 80.53 | CP109857.1 | TGTGTTGTGGACGGCCCGCAGCCATGGTGGGTTTCGACATCCGCGCGAAATGGGGCAAGCTGAAGTCGAGGGTTTTCTGACCATGCTCGCCACCGAGAAGCAAGTGGCGCCGGCCACCCACCGGCAGGCGCTCAACGCGCTGTTGTTCTTGTATCGGCAGGTGCTGGGCATGGAATTGCCGTGGATGCAGCAGATTGGTCGGCCGCCAGAACGCAAGCGGATTCCGGTGGTGCTGACGGTGCAGGAGGTTCAGACGTTGCTTTCGCACATGGCGGGCACCGAAGCGCTGTTGGCCGCCCTGCTTTACGGCAGTGGGTTGCGCCTGCGCGAAGCGCTGGGCCTGCGGGTCAAGGATGTGGATTTCGACCGCCACGCGATCATTGTGCGCAGCGGCAAGGGCGACAAGGACCGCGTGGTGATGCTGCCCAGGGCGCTCGTACCTCGGTTGCGGGCGCAGCTGATTCAGGTCCGCGCTGTGTGGGGGCAGGACCGTGCCACGG |
| *ermB* | 500 | 0.99 | 94.60 | CP131803.1 | ACAGGTAACGTCTATTGAATTAGACAGTCATCTATTCAACTTATCGTCAGAGCGATTAAAACTGAATACTCGTGTCACTTTAATTCACCAAGATATTCTACAGTTTCAATTCCCTAACAGACAGAGGTATAGCTTGTTGGGAATATTCCTTACCATTTAAGCACACAGCGTACGCACGCAGTGGTCGCTGACGAGCCGTGCGTCTGACATCTATCTGATTGTTGAAGAAGGATTCTACAAGCGTACCTTGGATATTCACCGAACACTAGGGTTGTTCTTGCACACTCAAGTCTCGATTCAGCAATTGCTTAAGCTGCCAGCGGAATGCTTTCATCCTAAACCAAAAGTAAACAGTGTCTTAATAAAACTTACCCGCCATACCACAGATGTTCCAGATAAATATTGGAAGCTATATACGTACTCTGTCTCAGCATGGGTCAATCGAGAATATCGTCAACTGTTTACTACGCATCAGTTTCATCAAGCAATGAAACACGCCA |
| *sul1* | 250 | 0.99 | 94.00 | NG_048096.1 | ACTGTCAAGCTTGGATCAGACGTCGTGGATGTCGGACCGGCCGCCAGCCATCCGGACGCGAGGCCTGTATCGCCGGCCGATGAGATCAGACGTATTGCGCCGCTCTTAGACGCCCTGTCCGATCAGATGCACCGTGTTTCAATCGACAGCTTCCAACCGGAAACCCAGCGCTATGCGCTCAAGCGCGGCGTGGGCTACCTGAACGATATCCAAGGATTTCCTGACCCTGCGCTCTATCCAAGCTTACTGTC |
| *tet33* | 500 | 0.99 | 86.2 | CP049865.1 | AACGTCGGAGTGCTGATCGCGCTCTACGCGGTAATGCAGTTCATCTTTGCCCCCGTACTGGGAACACTGTCGGACCGATTCGGCCGCCGCCGGGTGCTGCTTGTTTCCCTGGCCGGTGCGACCGTCGACTATCTCGTGCTCGCCACGACGTCCGCTCTGTCGGTGTTCTATATCGCCCGCGCAGTGGCTGGGATAACCGGAGCGACCAATGCGGTCACCGCCACCGTGATCGCCGACATCACGCCACCCCACCAGCGCGCCAAGCGTTTCGGTTTACTCAGTGCCTGCTATGGCGGCGGAATGATCGCGGGGCCAGCCATGGGTGGACTGTTCGGTGCCATCTCGCCACATCTGCCGTTTTTGCTCGCTGCTCTTCTCTCAGCGAGCAATCTGGCACTCACCTTTATCCTGTTACGCGAGACCCGTCCTGATTCGCCTGCGCGCTCTGCGTCGCTCGCTCAGCATCGTGGTCGCCCCGGCCTCAGCGCGGTGCCTGGGAT |
| *tetG* | 305 | 0.99 | 74.76^a^ | CP034668.1 | ACTGTCAAGCTTAGCCAATACTGGTTGAGGCCGCGAGCGACAAACCAACGGTCGCGGTGTTCCACTGAAAACGGTCCTCGCCATATATGACCCATAGGGCTGCAGGCACTTGGCCGATCAGTTGAATAATGAAGAAAACTGCGAAAAGCGCACCTAGCCCGCGCAATGCATCATCCAGCCGTAACAGAACGAATGGTTTGATGCGAACCGGCTTTCCGGTCCCGCCATGGCTGTGATGAGTCTCCTTGAGGAAAATGCAGGCAAGCAGGAACGCGATCCTTCTTCAACAATCAGAAGCTTACTGTC |
| *tetM* | 250 | 0.99 | 94.70 | CP017110.1 | ACTGTCAAGCTTAAACTACCTTAACAGAAAGCTTATTATATAACAGTGGAGCGATTACAGAATTAGGAAGCGTGGACAAAGGTACAACGAGGACGGATAATACGCTTTTAGAACGTCAGAGAGGAATTACAATTCAGACAGGAATAACCTCTTTTCAGTGGGAAAATACGAAGGTGAACATCATAGACACGCCAGGACATATGGATTTCTTAGCAGAAGTATATCGTTCATTATCAGTTAAGCTTACTGTC |
| *tetX* | 250 | 0.99 | 87.00 | CP114023.1 | ACTGTCAAGCTTTTATTATTAGGATTCGCAAATAATAAATTACCTTGATGAGCAGCCATTAGCCGGTTTCCATTGCATAGCTGAAAAAATCCAGGACAGTTCACCTCTGGATGATGAATATCGGCTTGTATATTGAAAGTACCTGTTTCTTCAACTTCCGTGTCGGTAACAAATTTTCTTACTTTAGACATTCCACCATTGGCAATAATAACCAGATCTGCTGTTTCACTCGGTTTATTAAGCTTACTGTC |
| *gfp* | 500 | 0.99 | 88.00 | EGFP_U57607 | AGCGGATAACAATTCCCCTCTAGAAATAATTTTGTTTAACTTTAAGAAGGAGATATACCATGGTGAGCAAGGGCGAGGAGCTGTTCACCGGGGTGGTGCCCATCCTGGTCGAGCTGGACGGCGACGTAAACGGCCACAAGTTCAGCGTGTCCGGCGAGGGCGAGGGCGATGCCACCTACGGCAAGCTGACCCTGAAGTTCATCTGCACCACCGGCAAGCTGCCCGTGCCCTGGCCCACCCTCGTGACCACCCTGACCTACGGCGTGCAGTGCTTCAGCCGCTACCCCGACCACATGAAGCAGCACGACTTCTTCAAGTCCGCCATGCCCGAAGGCTACGTCCAGGAGCGCACCATCTTCTTCAAGGACGACGGCAACTACAAGACCCGCGCCGAGGTGAAGTTCGAGGGCGACACCCTGGTGAACCGCATCGAGCTGAAGGGCATCGACTTCAAGGAGGACGGCAACATCCTGGGGCACAAGCTGGAGTACAACTACAAC |

^a^ The amplification efficiency for *tetG* (74.76%) falls below the standard acceptable range. The high coefficient of determination (r²=0.99) confirms reliable linearity of the standard curve; however, absolute *tetG* gene copy numbers may be modestly underestimated. Within-group comparisons for *tetG* remain internally valid as the same standard curve was applied consistently across all samples.

**Supplemental Table S3.** Thermocycling Conditions.

| Targets | Gene | PCR Recipe | Thermocycling Conditions |
| --- | --- | --- | --- |
| 16s  V3-V4 region | 16S rRNA | 2.0 µL of DNA (1 – 100 ng), 10.0 µL of Sso Advanced Universal SYBR Green Supermix (BioRad), 0.5 µL of each primer (forward and reverse at 10 µM), and PCR grade water to bring the total reaction volume to 20 µL. | 1 cycle of 95°C for 3 min; 40 cycles of 95°C for 30 sec, Annealing temperature for 1 min, Melt-curve analysis: 60°C to 95°C, 0.2°C/ read |
| Macrolides | *ermB* |  |  |
| Integron-Integrase | *intI1* |  |  |
|  | *intI2* |  |  |
|  | *intI3* |  |  |
| Sulfonamide | *sul1* |  |  |
| Tetracycline | *tet33* |  |  |
|  | *tetG* |  |  |
|  | *tetM* |  |  |
|  | *tetX* |  |  |
| GFP | *gfp* |  |  |

**Supplemental Table S4.** Ct values of Negative controls and Limits of quantification (LOQ) for 16S rRNA and resistance genes extracted from manure.

| Gene | Negative control  (Mean Ct values) | Stdev | LOQ  (Lowest Standard concentration)  (Mean Ct values) | Stdev |
| --- | --- | --- | --- | --- |
| *intI1* | 36.71 | 1.62 | 26.88 | 1.88 |
| *intI2* | No amplification | - | 27.92 | 0.35 |
| *intI3* | 34.90 | 0.39 | 27.82 | 0.59 |
| *ermB* | No amplification | - | 27.07 | 1.40 |
| *sul1* | No amplification | - | 28.14 | 0.54 |
| *tet33* | No amplification | - | 30.74 | 0.38 |
| *tetG* | No amplification | - | 30.47 | 0.35 |
| *tetM* | No amplification | - | 30.87^`^ | 1.87 |
| *tetX* | No amplification | - | 29.88 | 0.18 |
| *gfp* | No amplification | - | 32.67 | 0.40 |

**Supplemental Table S5.** DNA yield and quality metrics for all iDNA and exDNA extractions.

| Manure type | DNA type | exDNA type | Replicate | Yield_ug_g^b^  (Measured by Qubit) | DNA quality^a^ 260/280  (Measured by Nanodrop 2000) |
| --- | --- | --- | --- | --- | --- |
| Pit | exDNA | Free | 1 | 2.0375 | 1.6 |
| Pit | exDNA | Free | 2 | 2.8125 | 1.66 |
| Pit | exDNA | Free | 3 | 1.3 | 1.62 |
| Pit | exDNA | Weakly bound | 1 | 1.0375 | 1.68 |
| Pit | exDNA | Weakly bound | 2 | 0.7625 | 1.63 |
| Pit | exDNA | Weakly bound | 3 | 0.7875 | 1.62 |
| Pit | exDNA | Tightly bound | 1 | 1.3375 | 1.67 |
| Pit | exDNA | Tightly bound | 2 | 0.85 | 1.61 |
| Pit | exDNA | Tightly bound | 3 | 0.85 | 1.63 |
| Pit | iDNA | N/A | 1 | 32.125 | 1.81 |
| Pit | iDNA | N/A | 2 | 23.875 | 1.84 |
| Pit | iDNA | N/A | 3 | 28.775 | 1.81 |
| Fresh | exDNA | Free | 1 | 2.6375 | 1.62 |
| Fresh | exDNA | Free | 2 | 2.5625 | 1.69 |
| Fresh | exDNA | Free | 3 | 6.3875 | 1.61 |
| Fresh | exDNA | Weakly bound | 1 | 10.4125 | 1.65 |
| Fresh | exDNA | Weakly bound | 2 | 3.9875 | 1.65 |
| Fresh | exDNA | Weakly bound | 3 | 9.5 | 1.68 |
| Fresh | exDNA | Tightly bound | 1 | 1.6625 | 1.6 |
| Fresh | exDNA | Tightly bound | 2 | 2.3625 | 1.66 |
| Fresh | exDNA | Tightly bound | 3 | 1.8375 | 1.65 |
| Fresh | iDNA | N/A | 1 | 64.75 | 1.83 |
| Fresh | iDNA | N/A | 2 | 68.325 | 1.83 |
| Fresh | iDNA | N/A | 3 | 55.75 | 1.84 |

^a^ Nanodrop 260/280 ratios ranged from 1.6 to 1.84 across samples, with some values below the ideal range of 1.8–2.0, which may indicate residual protein or humic acid co-extraction common in complex manure matrices. ^b^Qubit dsDNA HS concentrations confirmed sufficient DNA yield for downstream qPCR and amplicon sequencing applications


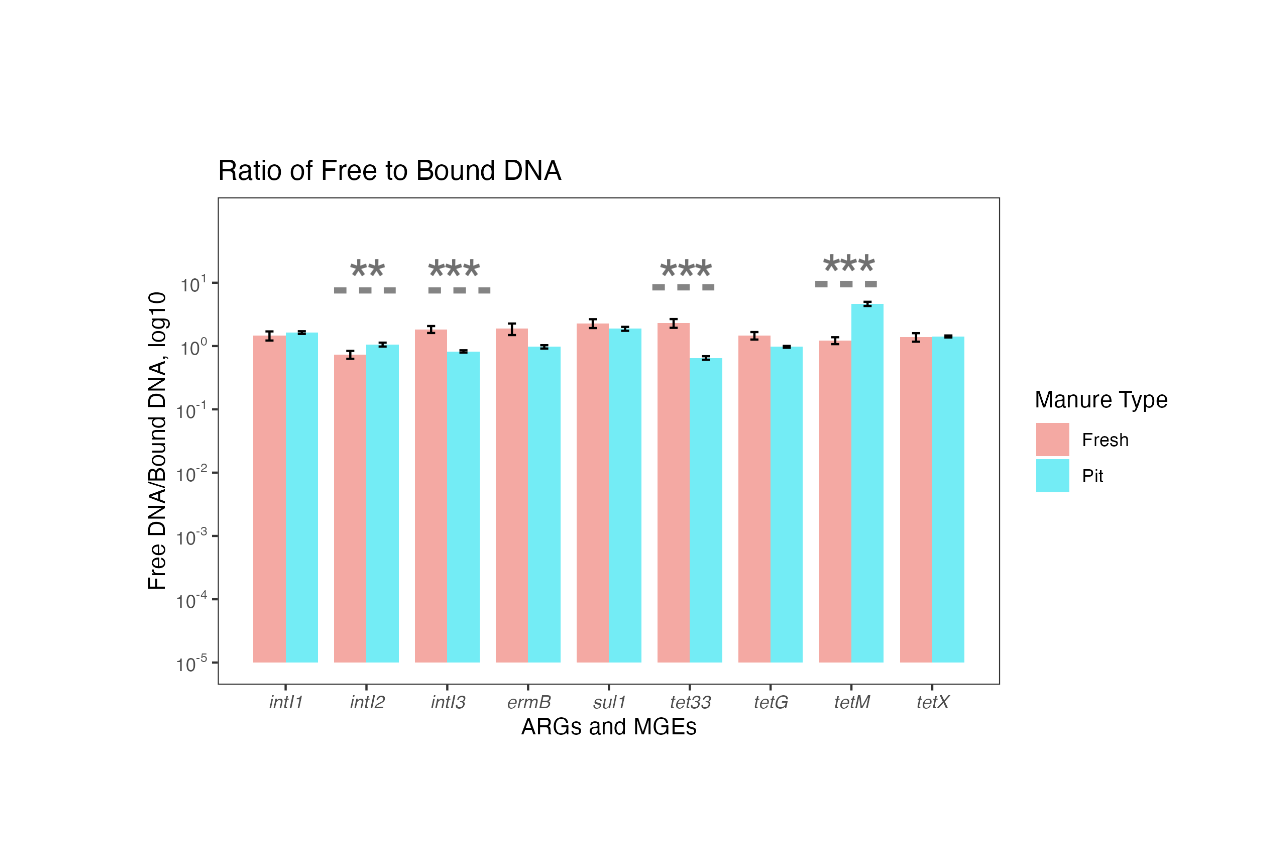


**Supplemental Figure S1.** Variations of ARGs and MGEs across exDNA fractions. (C) Bar plot showing the ratio of free to total bound (tightly and weakly) DNA in log-scale. Pairwise significance for manure types for each gene in the ratio was determined by Wilcoxon sum rank test. Asterisks indicate statistically significant correlations of ***p* < 0.01 and ****p* < 0.001, respectively. Error bars represent standard errors of the means (Mean ± SE).


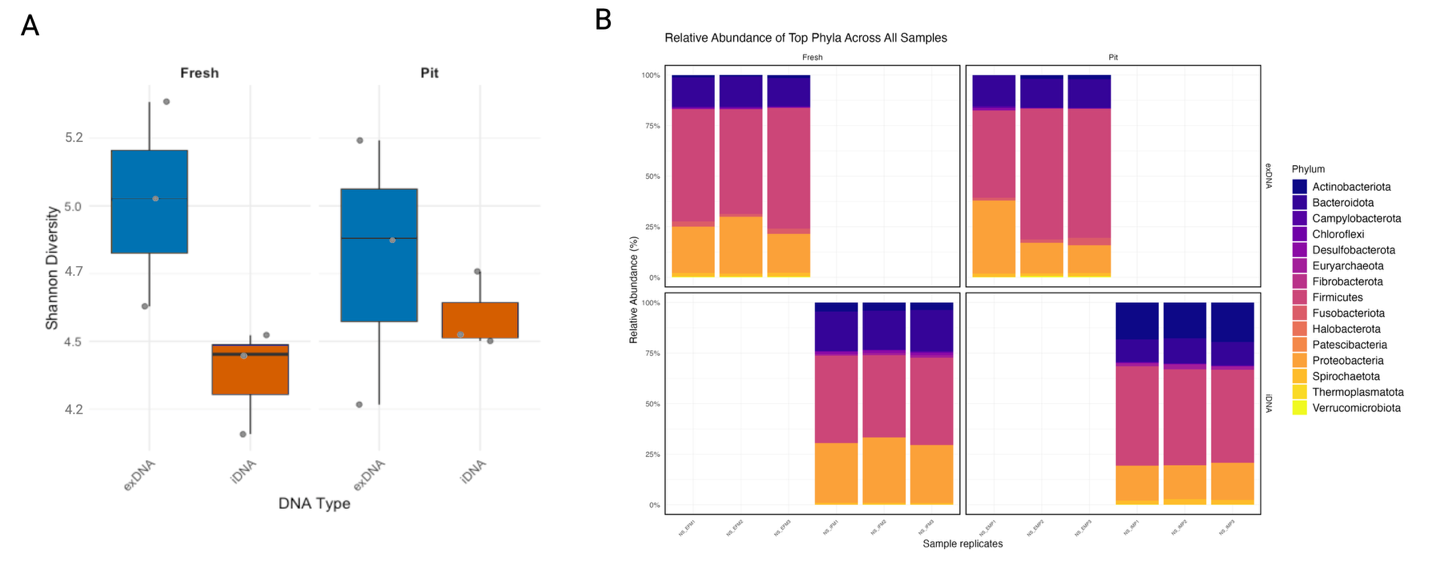


**Supplemental Figure S2.** Microbial community analysis shows A) Box plot of alpha diversity using Shannon’s H by sample type. Points indicate each individual sample measured. Significance determined by Wilcoxon sum rank test. Data not statistically significant (*p*>0.05). B) Relative abundance of bacterial phyla in exDNA and iDNA.


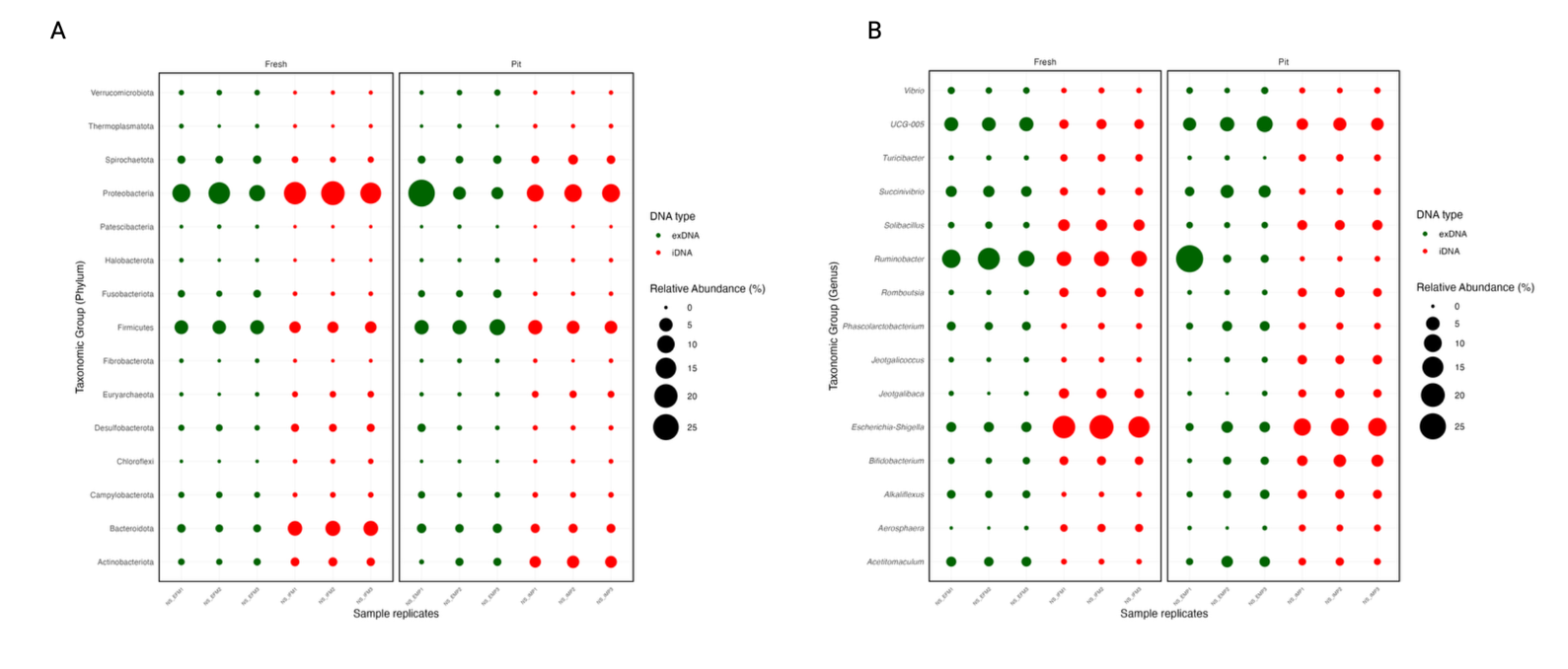


**Supplemental Figure S3.** Bubble chart showing the relative abundance of taxonomic groups in exDNA and iDNA. (A) Phylum-level abundance in fresh and pit manure samples. (B) Genus-level abundance in fresh and pit manure samples. Green bubbles represent exDNA, and red bubbles represent iDNA. The relative abundance equals the number of reads identified for a specific species by the total number of reads. Significant clustering was analyzed by PERMANOVA (*p* = 0.001, R² = 63.96%).

References

1. Nagler M, Podmirseg SM, Griffith GW, Insam H, Ascher-Jenull J. 2018. The use of extracellular DNA as a proxy for specific microbial activity. Appl Microbiol Biotechnol 102:2885–2898.

2. Stedtfeld RD, Guo X, Stedtfeld TM, Sheng H, Williams MR, Hauschild K, Gunturu S, Tift L, Wang F, Howe A, Chai B, Yin D, Cole JR, Tiedje JM, Hashsham SA. 2018. Primer set 2.0 for highly parallel qPCR array targeting antibiotic resistance genes and mobile genetic elements. FEMS Microbiol Ecol 94:fiy130.
